# Laryngeal Crisis or Potassium Iodide Poisoning?

**Published:** 1909-01-30

**Authors:** 


### Medicine.

### LARYNGEAL CRISIS OR POTASSIUM IODIDE POISONING?

The following case is possibly an instance of extreme susceptibility to iodide of potassium.

A man of 53 came under observation complaining of difficulty in walking. The trouble had begun two years before with curious sensations in his feet accompanied by such increased sensitiveness of the soles that he found it difficult to walk on uneven ground, and he could not go up a ladder at all because the rungs caused him so much pain. Gradually he walked less and less steadily, and there were typical lightning pains which he himself compared to sudden and violent electric shocks. Each shooting pain lasted barely a second, recurring at intervals of a few minutes and continuing thus for two or three days or a week at a stretch, and then disappearing for a fortnight or more before returning as before.

He also had a continuous sensation of gripping round the thorax "as though the chest were being squeezed between boards." For six months past there had been rapid diminution in his visual acuity, everything appearing as in a mist. It turned out that he had had a hard chancre when he was 25, without any secondary symptoms whatever. He exhibited Romberg's sign typically; the knee-jerks were absent, and there were Argyll-Robertson pupils.

On March 5 four doses of 15 grains each of iodide of potassium were prescribed. On March 6 the patient suffered from running at the nose and eyes, violent frontal and supra-orbital headache, and slight sore throat. The left eyelids were œdematous and there was a little reddening and swelling of the skin round them. There was an increasing œdema of the conjunctivæ with reddening which soon amounted almost to chemosis. The first impression given by the case was that erysipelas had set in; but there was no raised red border, no local heat of the parts, and no general pyrexia. The next impression was that the trouble was due to iodism, and the patient's own impression was that the medicine had caused these symptoms, so much so that he had not taken the full dose prescribed.

The potassium iodide was stopped, and 24 hours later the lesions were all much better. On March 7 four grains of potassium iodide were given. On March 8, in the absence of untoward symptoms, eight grains. On the 9th, 12 grains. On the 10th and 11th, 15 grains each day. On the 12th some dryness of the throat was complained of and the voice was husky. Examination showed no reddening nor other evidence of inflammation, but the epiglottis was moderately swollen and œdematous. The patient was not subject to sore throats; indeed he had never had one before that he could remember. A gargle was ordered and the 15 grains of iodide per diem were continued.

On March 13 some very alarming symptoms set in. Dyspnœa was observed and the patient complained of a feeling of "something in the throat." Nothing very urgent occurred, however, until 8 P.M., when the difficulty in breathing increased rapidly. The man was found seated in a chair by the side of his bed, with haggard eyes, deeply cyanosed face, dilating nostrils; he was clutching hold of adjacent objects and panting to get breath, with long inspiratory stridor and short silent expiration. There was marked sucking in above and below the clavicles and in the lower intercostal spaces. He was speechless, and from time to time made convulsive movements with his hands towards his throat to indicate that he felt he was being strangled. Mustard leaves to the chest and a hypodermic injection of morphia brought transient relief, but within a short while all the symptoms had returned in full degree. Tracheotomy seemed inevitable and it was performed forthwith.

It was then possible to make a thorough laryngoscopic examination. Marked œdema of the aryteno-epiglottidean folds without inflammatory reddening could be seen with ease; the swelling was so great that the cords could not be made out at this time. Next day the œdema of the larynx was rather less, and it was ascertained that there was neither spasm nor paralysis of the cords. This of course does not prove that there had been no spasm of them the day before, but it would exclude syphilitic paralysis. Œdema of the larynx was certain, spasm was possible, but there was no paralysis.

An attempt was made to leave the tracheotomy tube out on the 17th, but it had to be replaced after a time. It was not until the 20th that the tube was ultimately left out, and it was not until the 29th that the arytenoid swellings had completely gone. Needless to say potassium iodide had been discontinued from the first.

Throughout the attack there had been no pyrexia nor other evidence of microbial infection. By April 5 the patient was restored to his original condition of locomotor ataxy without any sign of laryngeal trouble.

---

What is the explanation of the laryngeal symptoms in this case? Might they be due to a tabetic laryngeal crisis? It is noteworthy that no so-called laryngeal crisis seems to have been recorded in a case of tabes in which no iodide of potassium has been given. This raises the suspicion that these particular crises may really be œdema laryngis due to the drug. It is further noteworthy that there was no recurrence, no second laryngeal crisis, in the above case; the symptoms disappeared entirely when potassium iodide was stopped.

Moreover, the larynx was not the only part to swell. Both conjunctivæ, one set of eyelids, and the tissues around the latter, had also been swollen up without being inflamed. This œdema also had disappeared when the drug was stopped.

It has long been recognised that iodide of potassium, at any rate if not free from impurities, is quite capable of producing œdematous infiltration of certain loose cellular tissues, especially in the eyelids, conjunctiva, epiglottis, and aryteno-epiglottidean folds. The liability to this is particularly great in elderly persons, especially if there is any degree of arterial degeneration or of granular kidney. Analyses of the drug have shown that the liability to accidents is much greater when iodate, nitrate, sulphate, and carbonate of potassium are present as well as iodide. The toxicity of potassium salts in some persons is well enough known. It seems likely that the case described above was one of acute œdema of the larynx due to the administration of impure iodide of potassium to an elderly tabetic whose kidneys may not have been quite sound, and it suggests the possibility that this is also the real nature of the so-called laryngeal crises of tabes dorsalis.

